# Antioxidant Polymeric and Non-Polymeric Nanoformulations for the Treatment of Autoimmune Diseases

**DOI:** 10.3390/cimb48060557

**Published:** 2026-05-26

**Authors:** Michail Varras, Fani-Niki Varra, Viktoria-Konstantina Varra, Panagiotis Theodosis-Nobelos

**Affiliations:** 1 Fourth Department of Obstetrics and Gynecology, ‘Elena Venizelou’ General Maternity Hospital, 11521 Athens, Greece; mnvarras@otenet.gr; 2 Department of Pharmacy, School of Health Sciences, Frederick University, Nicosia 1036, Cyprus; fannyvarra@gmail.com; 3 Medical School, Democritus University of Thrace, 68100 Alexandroupolis, Greece; 4 Department of Pharmacy, School of Health Sciences, University of Patras, 26504 Patra, Greece; nadv.ars2016@gmail.com; 5 Division of Aesthetics and Cosmetic Science, Department of Biomedical Sciences, School of Health and Welfare Sciences, University of West Attica, 12243 Athens, Greece

**Keywords:** autoimmune diseases, antioxidant nanoformulations, polymeric nanoparticles, oxidative stress, inflammation, targeted drug delivery, nanomedicine

## Abstract

Autoimmune diseases are characterized by chronic inflammation, immune dysregulation, and excessive oxidative stress, which collectively contribute to a progressive tissue damage and organ dysfunction. Although conventional immunosuppressive and anti-inflammatory therapies remain the main therapeutic approach, their clinical efficacy is often limited by poor pharmacokinetic properties, low tissue selectivity, systemic toxicity, and adverse effects following long-term administration. In this context, antioxidant-based nanoformulations have emerged as promising multi-target therapeutic strategies for the modulation of oxidative and inflammatory pathways involved in autoimmune disorders. This review focuses on polymeric and non-polymeric nanoformulations designed to improve the solubility, stability, bioavailability, controlled release, and targeted delivery of antioxidant and anti-inflammatory agents for autoimmune disease treatment. Recent advances in nanocarrier systems applications, including nanogels, poly(lactic-co-glycolic acid) (PLGA), polyethylene glycol (PEG), polymethacrylate, chitosan, hyaluronic acid, hydroxyapatite (HAP), lipid-based and ROS-responsive nanosystems, are discussed. The therapeutic potential of nanoencapsulated steroidal and non-steroidal anti-inflammatory drugs, antioxidant compounds, enzymes, inorganic elements, and nucleic acid-binding systems is evaluated through preclinical and limited clinical evidence. Many of these reported nanoformulations exhibit enhanced therapeutic efficacy, improved tissue targeting, reduced systemic toxicity, and the ability to simultaneously modulate oxidative stress and inflammatory signaling pathways. Despite the encouraging findings, important challenges remain regarding clinical translation, long-term safety, reproducibility, and large-scale production. In overall, antioxidant nanoformulations represent a promising and evolving platform for the development of more effective and targeted therapies against autoimmune diseases.

## 1. Introduction

Immune system comprises an essential part for the homeostasis of the organisms. However, auto-antigen identification may lead to autoimmunity and attraction of macrophages, T-lymphocytes, and the formation of immune complexes, as is the case with rheumatoid arthritis (RA), multiple sclerosis (MS), and systemic lupus erythematosus (SLE), leading to severe and potentially life-long damage and pathophysiological conditions that vary according to the type of the hypersensitivity reaction [1,2]. This is the case even at allografts rejection, due to immune responses, in case of end-stage organ failure.

Reactive oxygen and nitrogen species (ROS and RNS), as well as sulfur and chlorine species (RSS and RCS) production, is associated with inflammatory responses and phagocytes activation and constitutes an important defense mechanism, but in case of oxidative stressful conditions, like autoimmune inflammation and degenerative disorders, excessive generation of ROS takes place, resulting in the damage of cellular components as lipids, proteins, carbohydrates, and DNA [3,4]. Thus, a central feature of autoimmune diseases is the complex interplay between oxidative stress, chronic inflammation, and dysregulated immune signaling pathways [5]. Excessive production of reactive species apart from direct oxidative damage, modulates also multiple redox-sensitive molecular cascades involved in immune-cell activation and inflammatory progression. Among these, the nuclear factor erythroid 2-related factor 2 (Nrf2) pathway constitutes one of the major endogenous antioxidant defense mechanisms, regulating the expression of antioxidant and cytoprotective enzymes, including heme oxygenase-1 (HO-1), superoxide dismutase (SOD), catalase, and glutathione-associated enzymes [6,7]. In contrast, persistent oxidative stress may activate nuclear factor-kappa B (NF-κB), a transcription factor critically involved in the expression of pro-inflammatory cytokines, chemokines, adhesion molecules, and inflammatory mediators associated with autoimmune and auto-inflammatory diseases pathology [8]. Moreover, Toll-like receptor (TLR)-mediated signaling contributes to innate immune activation through the recognition of endogenous damage-associated molecular patterns (DAMPs), thereby amplifying inflammatory responses and cytokine production [9]. Dysregulation of Janus kinase/signal transducer and activator of transcription (JAK/STAT) signaling pathways has also been strongly implicated in abnormal cytokine signaling, immune-cell differentiation, and autoimmune tissue injury [10]. Importantly, these signaling pathways are highly interconnected, with oxidative stress acting both as a trigger and as a consequence of the inflammatory activation, thus sustaining a self-perpetuating cycle of immune dysregulation and chronic tissue damage [11]. Thus, reactive species are present in autoimmune diseases like the inflamed joint and lead to the progression of inflammation in a bidirectional manner, whilst antioxidant molecules have shown to be able, in an enzymatic and non-enzymatic manner, to adjust both the oxidative stress and the inflammatory processes [12,13]. Consequently, therapeutic approaches capable of simultaneously modulating oxidative stress and multiple inflammatory pathways may provide superior efficacy compared with conventional single-target therapies.

The most generally applied solution to these diseases is the usage of immunosuppressant drugs for these hypersensitivity reactions and rejection processes. However, adverse effects of these drugs, like hypofunction of the immune system, toxicity of various organs, cardiovascular defects and infections or tumor progression, render their application problematic, whilst difficulties in their pharmacokinetic profile, including reduced absorption, uncontrolled distribution and tissue targeting-membrane permeability reasons add towards this direction [14,15].

In this context, nanoformulations may be promising multifunctional delivery systems capable of improving the therapeutic performance of antioxidant and immunomodulatory agents in autoimmune diseases, as is the case with other diseases [16]. Polymeric and non-polymeric nanoparticles can significantly enhance drug solubility, chemical stability, bioavailability, tissue penetration, and controlled release, while simultaneously reducing systemic toxicity and nonspecific biodistribution, with improved internalization procedures and delayed clearance [17]. Their physicochemical characteristics, including particle size, surface charge, and surface functionalization, can be rationally engineered to facilitate selective targeting of inflamed tissues, activated immune cells, or oxidative microenvironments [18]. Furthermore, nanocarriers may exploit passive targeting mechanisms associated with enhanced vascular permeability at inflammatory sites, as well as active targeting approaches through the incorporation of ligands directed against overexpressed cellular receptors. Importantly, nanoformulations can also be designed as stimuli-responsive systems capable of releasing therapeutic agents under specific pathological conditions, including oxidative stress, acidic pH, enzymatic activation, or inflammatory signaling [19]. Such multifunctional nanosystems may simultaneously deliver antioxidants, anti-inflammatory drugs and macromolecules as nucleic acids and peptides, thereby enabling synergistic modulation of multiple pathways, including Nrf2, NF-κB, TLR, and JAK/STAT signaling cascades. Additionally, recent advances in nanomedicine have highlighted the potential of immunomodulatory strategies and biomimetic nanocarriers for precision immunomodulation and improved therapeutic selectivity in chronic inflammatory and autoimmune disorders, whilst nanoparticles (NPs) can also be loaded with multiple molecules for synergistic effects or amelioration of some of their adverse effects [20]. Taking all the above into account, and towards this direction, in this review, we are going to analyze polymeric and non-polymeric nano-formulations of direct and indirect antioxidant drugs (organic and inorganic) and enzymes, with anti-inflammatory potential, and in most of the cases a multi-targeting array of characteristics, that have been reported mainly during the last decade, alone or in combination with other compounds, for the control or the amelioration of autoimmune diseases (AID) and the antioxidant polymeric structures for AID.

## 2. Drugs with Antioxidant Status

Mycophenolate mofetil (MMF) and its active derivative mycophenolic acid (MPA) are effective treatments for autoimmune reactivity and especially against rejection of allografts. They are inhibitors of inosine-5′-monophosphate dehydrogenase, inhibiting T and B lymphocytes; however, attenuation of lipid peroxidation and decrease in the oxidant profile of inflammatory conditions, as the murine pleurisy in mice, shows its pleiotropic potential [21]. MPA is a hydrophobic drug with logP of 3.88, thus its aqueous solubilization and encapsulation is hindered rendering its applicability problematic. Thus, nanogels (NG) of MPA, for systemic lupus erythematosus treatment in mice, have been tested, elevating the survival time by 3 and 2 months, respectively, when used prophylactically or after the onset of the disease, whilst MPA alone was of no benefit since it was unable to internalize into the antigen presenting cells (APCs) [22]. The nanoparticles consist of biodegradable gel core of aminomethacrylate/succinylated β-cyclodextrin, combined with diacrylate-terminated coblock polymer of poly(lactic acid-*co*-ethylene glycol), with the usage of relevant initiator and remotely loaded on liposomes. After exposure to UV light, photopolymerization of the polyethylene glycol (PEG) monomers took place. Furthermore, CD4 antibody was incorporated on the nanogels for targeted delivery of the CD4 positive T cells that are abundant on the inflamed area in lupus, and are responsible for the stimulation of the autoreactive B cells [23]. The NG have the characteristics of liposomes and biodegradable polymers, with properties like encapsulation of a wide range of lipophilic and hydrophilic molecules, increased loading capacity, and stability. In vitro the NGs showed attenuation of the production of inflammatory cytokines, via dendritic cells function alteration and two-fold reduction in interferon-γ producing CD4 T cells, improving the already known anti-inflammatory potential of MPA.

Interestingly, in another case, MMF was used prophylactically for heart transplant rejection [24]. In pre-perfusion of the mice heart tissue with MMF, loaded on PEG-PLGA nanoparticles with solvent evaporation and centrifugation method, showed diminished transplant vasculopathy [as the low levels, compared to control group, of VCAM (vascular cell adhesion molecule 1) and P-selectin showed], being able to be up-taken by the tissue according to the human umbilical vein endothelial cells permeation test. The MMF-NPs offered good histological appearance of the heart even 28 days post-transplantation with no effect on the peripheral immune system, as is the spleen and the draining lymph nodes, potentially due to the controlled delivery of the drug.

Metronidazole (MTZ) is a bactericidal antibiotic with ambiguous in vitro antioxidant profile, acting also as a prooxidant in some cases. However, it seems to inhibit lipid peroxidation in indirect antioxidant pathways [25]. Metronidazole is used for the treatment of colon auto-immune diseases like Crohn’s disease, but its absorption from the upper part of the intestine makes its release in the colon difficult. Thus, MTZ microspheres, consisting of the pH sensitive Eudragit S 100, have been prepared for the targeted local release at the colon and increased stability and encapsulation efficiency for the treatment of inflammatory bowel disease (IBD) [26]. The microspheres were synthesized by mixing of the polymer, MTZ and magnesium stearate, followed by solvent evaporation of acetone and vacuum filtration of the microspheres that had good flow properties and encapsulation efficiency of around 68% and with controlled drug release suitable for the usage in IBD. At another preliminary research [27], dapsone (DAP, 4,40-diamino diphenylsulphone), another antibiotic, against leprosy, with antioxidant activity, lipid peroxidation, nitrotyrosine content, and myeloperoxidase inhibitory potency in ischemia-reperfusion, was tested [28]. DAP has shown to mitigate the activation of polymorphonuclear leukocytes with inhibitory potency against auto-immune conditions [29]. Rojo et al. in order to increase the solubility and improve the non-specific distribution of DAP, synthesized two polymeric derivatives [27]. These polymers were derived by the polymerization of the monomer dapsone methacrylamide (DapMA, synthesized by the reaction of dapsone with methacroylchloride) giving the homopolymer p(DapMA) or the polymerization of 2-hydroxyethyl methacrylate (HEMA) with DapMA, giving the relevant co-polymer. The synthesis of both polymers took place by free radical production with 2,2-azobisisobutyronitrile (AIBN). Thermal and size exclusion analysis showed high stability (higher for the homopolymer), with low hydrolysis rate of DAP, for both polymers, and in vitro NO inhibition assay gave insight for the anti-inflammatory, dose dependent, potency of the polymers, showing potential also for the sustained release of the active substance.

Teriflunomide (Ter) is an active immunomodulatory drug (derived by activation of leflunomide), blocking the enzyme dihydroorotate dehydrogenase, inhibiting of the de novo biosynthesis of pyrimidine nucleic acids, and exerting antioxidant and cytostatic effects that renders it a good anti-proliferator of immune cells for auto-immune demyelinating disorders, such as MS [30,31]. Nano-lipid carriers (NLC) of Ter, using biodegradable and biocompatible polymers, in a melt emulsification ultrasonication method, were synthesized for the optimization of the Ter administration intranasally (i.n.) [32]. The resulted NLC showed increased size and zeta potential (99.82 ± 1.36 nm and −22.29 ± 1.8 mV, respectively) with entrapment efficiency up to 83%, with permeability of Ter up to 830 μg/cm^2^ with the mucoadhesive NLCs (MANLC). Ter-NLCs were tested at cuprizone induced Wistar rats where TFM-NLC 100 μg/mL and TFM-MANLC 50 μg/mL, were given orally. Both groups showed normal behavior at the exteroceptive behavioral mode with reduced number of entries and movements in the open compartment and significant effect on demyelination, but with significant effect of TFM-MNLC i.n. compared to the oral TFM-NLC. Simultaneously, the nano-formulation provoked no gross changes in biomarkers and sub-acute toxicity (perhaps due to its improved pharmacokinetic behavior) when compared with control. Teriflunomide has been also applied in co-administration with methotrexate (MTX) incorporated in hydroxyapatite (HAP) nanoparticles for RA treatment [33]. HAP-NPs were prepared with wet chemical precipitation method using Ca (NO_3_)_2_.4H_2_O and (NH_4_H_2_PO_4_) as calcium and phosphate precursors, and ammonia and cetyltrimethylammonium bromide, and the final NPs were loaded with drugs by the mechanism of “sorption” (physical adsorption and absorption). The size of the optimized formulations, Ter-HAP-NP and MTX-HAP-NP, was found to be 224.3 ± 83.80 nm and 268.3 ± 73.86 nm with drug loading 53.11 ± 0.84% and 67.04 ± 1.12%, respectively, with sustained release pattern. In the in vivo complete Freund’s adjuvant (CFA) induced arthritis model, the combined formulation of MTX-Ter-HAP-NP could markedly restore the ankle-joint architecture, with a decrease in the diameter and arthritis score compared to the single compound or the non NPs administered compounds. MTX-Ter-HAP-NP also had remarkable decrease in serum GOT and GPT (glutamic oxaloacetic transaminase and glutamate pyruvate alanine aminotransferase) levels with less hepatocyte swelling and fibrous connective tissue proliferation compared to the methotrexate tablet oral administration. Co-administration of MTX and Ter with HAP-NP, could effectively act on RA at dose half of the tested with other formulations, accentuating its improved pharmacological and toxicological profile, perhaps through the improvement of the pharmacokinetic characteristics that led to their improved delivery.

***Steroidal and Non-Steroidal*** ***Anti-Inflammatories***

Oxidative stress and inflammation are interrelated and the appearance of one could lead to the promotion of the other [34]. Thus, in many cases, no distinct characteristics are sometimes obvious of which one was responsible for the cause of the other. Classical non-steroidal anti-inflammatory drugs (NSAIDs) inhibit both cyclooxygenase 1 and 2 (COX 1 and 2) leading to intermediate products of arachidonic acid, via radical and hydroperoxides formation, giving rise to ROS production, and in the case of co-existing inflammatory condition COXs, expression can be induced leading to oxidation of cellular mediators like glutathione and nitrogen monoxide, deregulating further the cell signaling, as well as the redox potential of the organism towards the oxidant conditions [35,36]. As a result, NSAIDs and some anti-inflammatory drugs could be effective towards the reversal of the oxidative stress that is driven by inflammation.

Tenoxicam (TNX), a classical NSAID, was incorporated in in situ formed implants and in situ forming microparticles (IMP), since the former have some issues concerning the increased burst release of the drug with toxicity concerns and variable implant shape [37]. For the preparation of the implants, different amounts of poly (DL-lactide) (PDL 02) and TNX were dissolved in N-methyl-2-pyrrolidone (NMP) with sonification and for the microparticles PDL 02, TNX (20 mg/mL) and PF68 surfactant were dissolved in NMP and the resulted polymeric solution was emulsified with sesame oil (SO) in various ratios for the formation of an oil in oil IMP system. The release studies and the histopathological studies showed that the optimized TNX microparticles consisted of 1 part of polymer and 4 parts of SO and polymer concentration of 30%. The TNX in situ microparticles were compared to oral administered TNX for FCA induced arthritic model in rats. IMP was of low burst release with higher and prolonged protection against inflammatory responses, with potential synergistic effect of the antioxidant and anti-inflammatory SO and TNX, resulting in statistically significant reduction in paw weight and paw oedema, with improved oxidative biomarkers [SOD, MDH (malondialdehyde), MPO (myeloperoxidase)] and with low liver biomarkers (similar to those of control after 21 days), compared to oral TNX, accentuating the low toxicity and the activity of the chosen IMP in the improvement of the activity of TNX.

Steroidal anti-inflammatory drugs are another part of the treatment of auto-immune diseases with long lasting history [38]. Betamethasone, a widely used glycocorticosteroid (GLC), has been encapsulated with poly(lactic acid), and tested for experimental, S-antigen peptide induced, experimental autoimmune uveoretinitis (EAU) [39]. Additionally, rhodamine encapsulated NPs were used together for visualization of the distribution by confocal microscopy. The NPs accumulated at the retina and the choroid and were preserved there for all the testing period (7 days), with reduction in the clinical and histological scores (Muller cells hypertrophy reduction) and the T cell and macrophage infiltration. The improvement in the clinical scores was more profound than the administration of betamethasone alone at the same dose as well as the histological scores, showing the improvement of the delivery behavior of betamethasone via its encapsulation. At another study, dexamethasone (DEX) NPs were used subconjunctivally for the replacement of intravitreal dexamethasone implants that may have serious adverse effects, like hemorrhage and endophthalmitis [40]. Since subconjunctival DEX sodium is highly water soluble, a sustained release encapsulation method was applied using carboxyl-terminated poly(lactic-co-glycolic acid) (PLGA) with divalent ionic bridging between DEX and the NP. The resulted mixture gave NPs with low diameter (210 nm) and almost zero surface charge, with sustained release ability for more than 3 weeks. As a result, reduced microglial activation was observed in the retina accompanied by decreased clinical scores at a rat model of EAU after a single dose of DEX-NPs.

Li et al. [41] have also used celastrol, a natural steroid, derived from root bark of Tripterigium wilfordii, (with anti-inflammatory potency, against auto-immune and chronic inflammatory diseases [42]), for the corneal neovascularization attenuation via mitigation of cytokine secretion and macrophage induction. Due to the low solubility of celestrol in the aqueous body fluids, celastrol-loaded poly(ethylene glycol)-block-poly(ε-caprolactone) (PEG-b-PCL) nanomicelles were prepared and administered with sub-conjunctival injection. As a result, the nanomicelles significantly inhibited the migration of human umbilical vein endothelial cells and the expression of TNF-α (tumor necrosis factor-alpha), VEGF (vascular endothelial growth factor) and various other inflammatory factors by the macrophages with decrease in the length and area of the corneal neovascularization.

**Figure 1:**
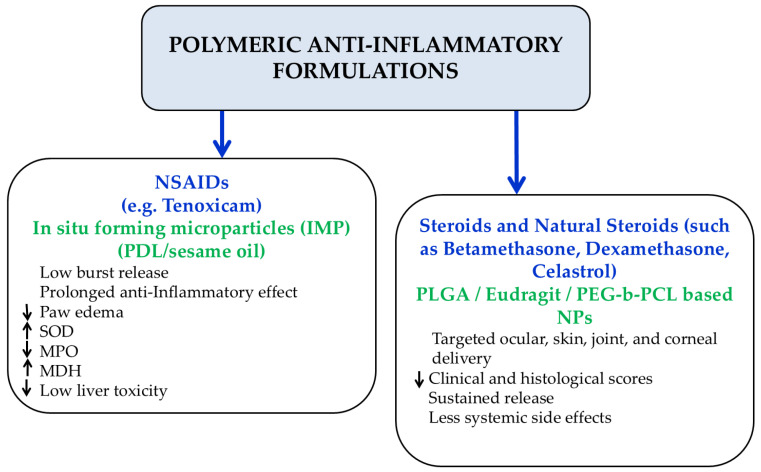
Polymeric NSAIDs and steroidal compounds as anti-inflammatory agents.

Dexamethasone (DEX) NPs has also shown improved controlled drug release on the skin with increased amount of drug being delivered into the epidermis, showing increased potential for dermatological autoimmune conditions amelioration [43]. In this case, cationic Eudragit^®^ RS 100 was used for adhesion and interaction increase with the viable epidermis, synthesized via mixing of the polymer with caprylic and capric triglycerides with DEX and injection of the mixture in aqueous solution of polysorbate 80 with subsequent evaporation of the organic solvent (acetone) and water. The resultant nanocapsules had mean particle size of 139 nm, positive zeta potential and increased (>80%) encapsulation efficiency, with formation of plastic behavior hydrogels with decreased penetration into dermis compared to epidermis, the target tissue. Similarly, in RA, DEX pH-sensitive delivery system composed of N-(2-hydroxypropyl)methacrylamide copolymer (P-Dex) was prepared for the selective release at the inflamed area, with the highest levels of drug release being observed at pH = 5 (pH < 6 is observed at the synovial fluid of RA joints) with higher and longer lasting anti-inflammatory activity than DEX alone and improved morphological preservation of the bone and cartilage [44] (Figure 1).

**Figure 2:**
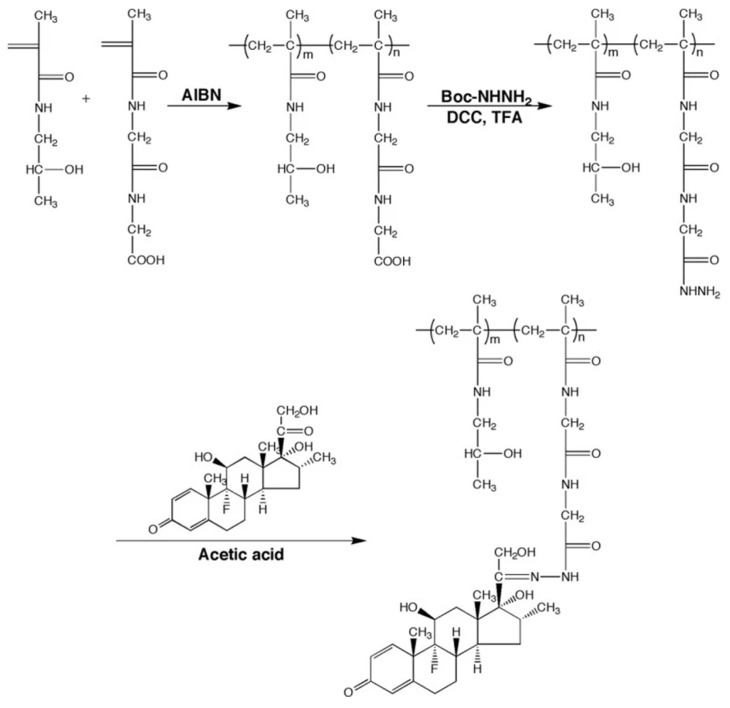
The synthesis of N-(2-hydroxypropyl)methacrylamide (HPMA) copolymer-dexamethasone conjugate. Conjugation to dexamethasone may occur at either the 3 or the 20 carbonyl group (an example of the latter is shown). TFA, trifluroacetic acid. Reprinted from ref. [44].

The synthesis of the NPs took place via co-polymerization of N-(2-hydroxypropyl)methacrylamide (HPMA) with N-methacryloylglycylglycine (MA-Gly-Gly-OH) using 2,2′-azobisisobutyronitrile initiator. The product amidated with Boc-hydrazine (carbazic acid tert-butyl ester) with DCC (N,N′-Dicyclohexylcarbodiimide) and after the Boc protection the resulted polymer was mixed with DEX and acetic acid as catalyzer for the final covalently bonded DEX-NPs (Figure 2).

**Figure 3:**
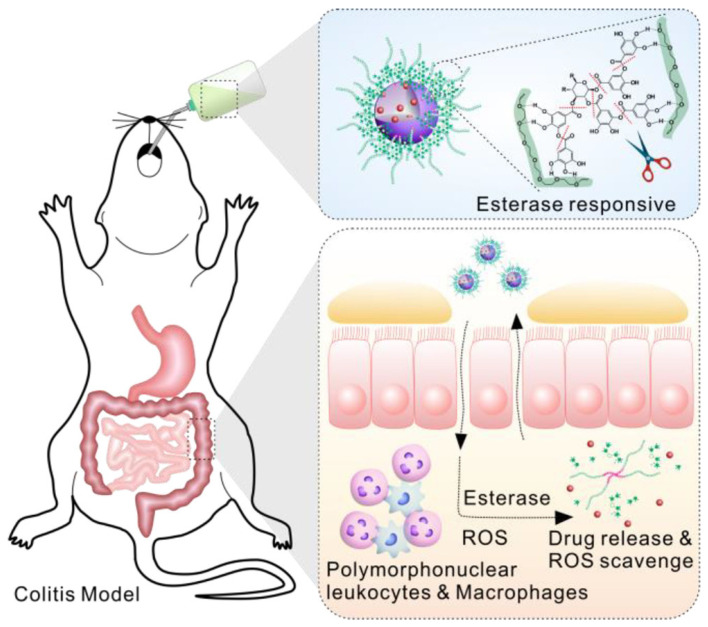
Schematic illustration of oral delivery of DEX-NPs. DEX-NPs remain stable in the environment of the gastrointestinal tract and noninflamed mucosal tissues; however, at sites of intestinal inflammation, where esterases and ROS are upregulated, DEX-NPs degrade, thus releasing DEX and scavenging ROS at the sites of inflammation (reprinted with permission from reference [46]. Copyright 2018 American Chemical Society).

DEX has also been used for IBD targeting, with a new drug delivery system composed of polyphenols (with antioxidant and anti-inflammatory properties [45]), with wet resistant adhesion and degradable properties, and PEG or the lipophilic F-127 or F-68 [46], by self-assembling via hydrogen bonds of the polyphenol with the polyethylene glycol moiety of the polymer. The final NPs could be degraded by esterases which are highly expressed at the IBD sites delivering the encapsulated DEX and the antioxidant catechol containing compounds of the polyphenols at the target inflamed area (Figure 3). After testing the Tyndall effect, it was found that the higher the hydrophobic chain the bigger the effect facilitating the micellar formation. These results together with the finding that the tannic acid (TA) derivatives had lower size and polydispersity index, led to the choice of TA and F-68 for further study with more uniform NPs. The DEX-NPs showed increased stability at the gastrointestinal environment with substantial increase in their release in the presence of esterases (three times more), with parallel ROS scavenging, dose dependent activity and excellent biocompatibility, as no cytotoxic effects were observed in mouse fibroblast cell lines. Furthermore, the negative potential of polyphenols offers high interaction potency with the inflamed mucosa since the colitis epithelium is positively charged due to specific peptides accumulation (bactericidal/permeability-increasing protein, and antimicrobial peptides) [47]. Thus, the NPs could effectively target colitis as the usage of fluorescence encapsulated dye showed, in comparison to the healthy colons. This explains and the weight gain of the colitis induced DEX-NPs oral treated compared to control group that showed 14% decrease in the body weight, resembling to the healthy control group. This effect was accompanied by reduction in myeloperoxidase and TNF-α activity and low neutrophil invasion with physiological epithelium structure in the treated mice.

## 3. Cellular Components Mimicking or Binding Polymers

Another category of polymers used against inflammatory conditions, such as systemic lupus erythematosus (SLE) are the nucleic acid binding polymers (nucleic acid scavengers, NAS). These polymers are cationically charged amine rich structures with ability to bind to self-nucleic acids (RNA and DNA), that are responsible for the elicitation of the innate immune response, by the activation of the endosomal Toll-like receptors (TLRs), leading to downstream activation of signaling and expression of pro-inflammatory cytokines, autoantibodies, and SLE progression [48,49,50,51,52,53]. Holl et al. used two strains of mouse models of SLE, with close resemblance to human SLE, to assess immune activation and resolution of inflammation in the presence of NASs [54]. Tape stripping, for short term treatment, was applied at the dorsal area of the mice and PAMAM-G3 (20 mg/kg s.c.), at the time of striping and every 3 days after. Whilst for the long-term treatment, mice with SLE were treated with PAMAM-G3 (20 mg/kg) twice a week for a period of 8–10 weeks, starting at 10 weeks of age. The dose of PAMAM-G3 was up to 10 fold decreased than the maximum tolerated dose (MTD) of PAMAM-G3 (100–200 mg/kg) [55]. PAMAM-G3 as a cationic dendrimeric polymer with 1,4-diaminobutane core, containing 32 surface amine groups [56], could significantly decrease disease grades in all the pathological categories, with skin damage recovery. This effect did not seem to be related to the immune cell recruitment (macrophages, neutrophils, and T cells) that remained unchanged; however, NASs blocked the nucleic acids derived TLRs activation, facilitating wound healing by extracellular nucleic acids scavenging, reducing TLR activation and sustained aberrant inflammation. Additionally, PAMAM-G3 treatment significantly reduces glomerulonephritis and kidney damage due to reduced deposition of complement C3c in the lupus-prone mice. These effects may at least partially derive by its impact on the levels of anti-nuclear and anti-DNA antibodies (Abs), with much less intense or even absent staining (fluorescent anti-nuclear Ab assay, ANA) intensity when serum from PAMAM-G3–treated lupus-prone mice was evaluated, compared to samples from phosphate buffer saline treated mice. Interestingly, treatment did not suppress the immune system, offering protection of mice from lethal influenza infection. Cell free nucleic acids serve as an important damage associated molecular pattern for immune reactivity in various auto-immune diseases, including RA [57]. Peng and his colleagues used another NAS polymer for cell-free deoxyribonucleic acid (cfDNA) scavenging [58]. The NPs were composed of linear polymer backbone (biodegradable polycaprolactone-PCL was chosen for toxicity reduction) and high-density grafted dendrons (cationic polyamidoamine-PAMAM). For their preparation, the PCL backbone with bromine groups was synthesized by the ring-opening polymerization of bromo-3-caprolactone, and subsequent transformation of bromine groups into azide. PAMAMs were protected via tert-butoxycarbonyl (BOC) and introduced into the azide polymeric backbone by a copper-catalyzed alkyne–azido click reaction, and at the final stage removal of the BOC group resulted in PCL-g-PAMAM dendronized polymers. Various degree of polymerization and generations of PAMAM dendrons were obtained, with the most promising concerning toxicity and the approach to the arthritic area in collagen induced arthritis (CIA) and the DNA binding, ability being the 384-G3 (the first number corresponds to the degree of polymerization of PCL and the latter to the generation number of the PAMAM Dendron), and with the anti-inflammatory and the TLR inhibition activity increasing with both the polymer and the side Dendron generation increase. The 384-G3 decreased very significantly (*p* < 0.001) the levels of TNF-α and IFN-α in peripheral blood mononuclear cell (PBMC) derived macrophages and at the synovial fluid derived resident macrophages after a 24-h co-culture with cfDNA. At CIA rats, joint swelling was developed from day 13, and from then daily intravenous injections of 384-G2 and 384-G3 were performed. The clinical severity of hindpaw and forepaw swelling was greatly inhibited and the joints showed severe hyperplasia of the synovial membrane and immune cell infiltration in multiple joints of the CIA model group, whilst treatment with 384-G2 and 384-G3 displayed almost normal synovial membranes in the hindpaws and the other joints. Specifically 384-G3, had better in vitro DNA binding (384-G3 treated groups showed lower cfDNA levels, similar to that of the control group), displayed better joint protection in the smaller toe joints that are mostly affected in RA, improving also the bone destruction, bone mineral density (BMD), bone surface area/bone volume (BSA/BV), and bone volume/total volume (BT/TV) of the model rat. Furthermore, body weight loss was negligible, with no observed organ damage and good biocompatibility, verifying and the good toxicological profile of the NPS.

Towards the direction of scavenging components of the serum that are related to the progression of the auto-immune diseases, glycopeptides, reproducing antigen recognizing antibodies, have been applied [59]. Thus, such probes that could interact with autoantibodies and biomarkers of these diseases could offer diagnostic, prognostic, and treatment options [60]. Branched PEG-based spacer polymers with addition of glycosylated asparagine anchors [peptide antigens, Asn(Glc)] have shown to offer affinity with anti-Asn(Glc) antibodies in Multiple Sclerosis (MS) [61]. Antibodies to anti-hyperglucosylated NTHi (non-typeable Haemophilus influenza, which was reported to express cell-surface adhesins including N-Glc) adhesion protein HMW1ct(N-Glc) (bearing multiple glycosylated epitopes on asparagine residues), were present in a subpopulation of MS patients [62]. Due to this, novel peptide-based dextran conjugates with NTHi HMW1ct(N-Glc) were prepared, and with the aim of developing shorter peptide probes, it was shown that the di-N-glucosylated adhesin peptide Ac-KAN(Glc)VTLN(Glc)TTNH_2_ could bind IgM antibodies in a similar to the parent glucosylated bacterial adhesion manner [63]. Thus, a series of shorter peptides and functionalization of dextran with glycidyl-propargyl was applied with final conjugation of the parent moieties via “click”-type chemistry with copper catalyzed alkyne-azide coupling reaction (CuAAC) [64]. The obtained final Dex40-Peptide with the Ac-KAN(Glc)VTLN(Glc)TTG-K(N_3_)-NH_2_ had 19.5% peptide loading on the dextran polymer and it had optimal capturing properties of both IgG and IgM antibodies, and similar to HMW1ct(N-Glc) antigen affinity, being a promising tool for selective identification and purification of high affinity, and of specific high affinity, autoantibodies from the serum of MS patients.

**Figure 4:**
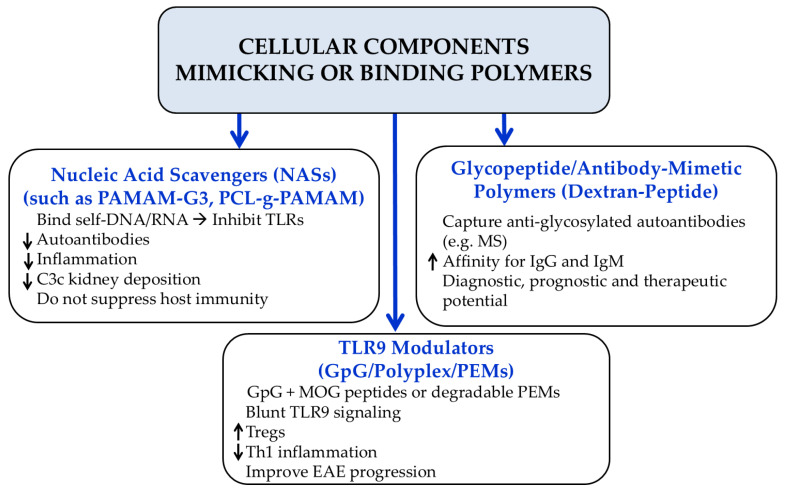
Cellular components mimicking or binding polymers and their therapeutic potential.

In view of TLR signaling inhibition and myelin oligodendrocyte glycoprotein (MOG) peptides, Hess et al. [65] used a DNA sequence called GpG (an analog of CpG, exhibiting a substitution of guanine for cytosine, unmethylated, single-stranded DNA with a phosphorothioate backbone that can bind TLR9 [66]), for the suppression of the proliferation of inflammatory TH1 cells and the attenuation of the experimental autoimmune encephalomyelitis (EAE). Further, enhanced induction of tolerance was succeeded by mixing GpG with MOG, modified with one or two cationic arginine residues (for ionic binding with the GpG), acting as myelin self-antigens, and polarizing T cells away from an inflammatory phenotype. The polyplex-like structures assembled by mixing GpG and a myelin peptide in various MOGRx/GpG ratios. The polyplexes could be up-taken by the antigen presenting cells, blunting TLR9 signaling and allowing the proliferation of MOG-specific T cells, towards the Tregs direction and away from Th1 cell formation, whilst the polymeric self-antigens corporation may lead to scavenging and clearance of these particles, resulting in activation of tolerance pathways that do not contain inflammatory cues [67]. Furthermore, they offer protection from enzymatic degradation, and tunable physicochemical properties, with reduction in TLR signaling, and in myelin-driven inflammation in dendritic and T cells, with improvement of the progression, severity, and incidence of MS. In another approach using only GpG oligonucleotide, Tostanoski et al. used degradable poly(β-amino ester) (Poly1) incubated with GpG to deposit the desired number of Poly1/GpG bilayers self-assembled into polyelectrolyte multilayers (PEMs) in order to offer slow release of cargo for inflammation regulation and immune cells tolerance promotion [68]. For the Poly1 synthesis, 4,4′-trimethylenepiperidine was mixed with 1,4-butanediol diacrylate at 50 °C for 16 h, and afterwards precipitation of the resultant polymer and lyophilization were followed. It was showed a time-dependent increase in the mass of nucleic acid release over three days. Poly1/GpG restrained primary dendritic cells CpG-induced activation for all defined time intervals of 1, 3, 5, or 7 days, indicating that released GpG maintains function after incorporation into degradable PEMs and following release for up to seven days. Additionally, in TLR9 signaling lines, the PEMs derived GpG treatment significantly decreased the activity compared to the non-PEMs, although administration into dendritic cells, did not seem to limit their proliferation, but offered increased anti-inflammatory activity with significant decrement in the IL-6 (interleukin-6) levels, also skewing T cell phenotype towards a tolerogenic population, enhanced by the incorporation into degradable PEMs (Figure 4).

Furthermore, Kong et al. [69] used phosphorylatable short peptide conjugated chitosan (pspCS) for the incorporation of plasmid DNA (pDNA) for the analysis of gene delivery transfection in inflammatory conditions such as the experimental autoimmune encephalomyelitis (EAE). Different N:P ratios (ratio of positive polymer amine groups to negative nucleic acid phosphate groups) were used to form pspCS/pDNA particles of different size and zeta potentials and transfected to astrocytes isolated from EAE. The highest cell permeability and transfection efficiency was performed by the larger-sized pspCS/pDNA particles (formed at lower N:P ratios), with zeta potential not being the important factor (although positive potential leads to better internalization), compared to the size. Cationic polymer/pDNA nanoparticles showed that internalization is achieved via endocytosis and phagocytosis, especially for advanced transfection efficiency of large pspCS/pDNA particles, rendering this technique a promising tool for the gene transfer strategies.

***Methotrexate*** 

Methotrexate (MTX) is a folate anti-metabolite, offering a reduction in systemic inflammation and tissue damage, via inhibition of purine and pyrimidine metabolism, transmethylation and induction of anti-inflammatory mediators [70,71]. Furthermore, MTX has shown to inhibit the formation of adducts, produced by aldehydes, from lipid peroxidation, and proteins, and scavenge ROS (especially superoxide radical), offering decrease in Nrf2 by ameliorating the oxidative stressful conditions in the surrounding area [72,73].

In a combined folate-receptor targeting and methotrexate loaded application, pH responsive nanοcarriers were used, composed of a hydrophilic polyethylene glycol (PEG)–poly(lactic-co-glycolic acid) (PLGA) outer core, covalently esterified with FO at the PEG moiety, whilst egg lipids formed the surface shell [74]. The inner shell was composed of the hydrophobic PLGA and the pH degradable poly(cyclohexane-1,4-diylacetone dimethylene ketal) (PCADK) [75] that encapsulated MTX. As expected, the lipid NPs had pH dependent release in vitro, with higher activity and uptake by the macrophages [due to the folate receptor β (FRβ) active targeting] than the non-FO lipid NPs, and their activity was confirmed on adjuvant-induced arthritis (AIA) rat model, after intravenous treatment with the tested NPs every two days, starting from the fourteenth day. Rats treated with FO-MTX-NPs showed statistically significantly (*p* < 0.01) lower RA clinical scores, than MTX alone or non-FO MTX-NPs, and the same was observed with the paw thickness, swelling or erythema testing. Furthermore, reduced bone and cartilage destruction and synovial hyperplasia were observed after histological and radiography testing of the joints. These results are also confirmed by statistically lower serum TNF-a and IL-6 levels compared to the other forms of treatments. For the preparation of the LPNPs, synthesis of FO-PEG-PLGA via esterification took place and the final product was prepared by an emulsion solvent evaporation technique by dissolution of PLGA, FO-PLGA-PEG, PCADK, and egg lipids and the addition of MTX under sonification [74]. The final mixture was added to the polyvinyl alcohol solution and emulsified. The final pellet product was collected after solvent evaporation and centrifugation. At another FO/MTX application, poly(amidoamine) (PAMAM) dendrimers, with uniformity, biocompatibility, defined structure and capability of coupling multiple molecular entities, were conjugated with FO and MTX, starting from successive Michael additions of methacrylate to ethylene diamine, and condensation reaction, resulting in generation of partially acetylated dendrimers that incorporate amidated FO, and esterified MTX, with glycosylated surface, for surface neutralization [76]. The dendrimers could be internalized in a FRβ specific manner, with reduction in histological and clinical scores, in collagen-induced arthritic rat model.

**Figure 5:**
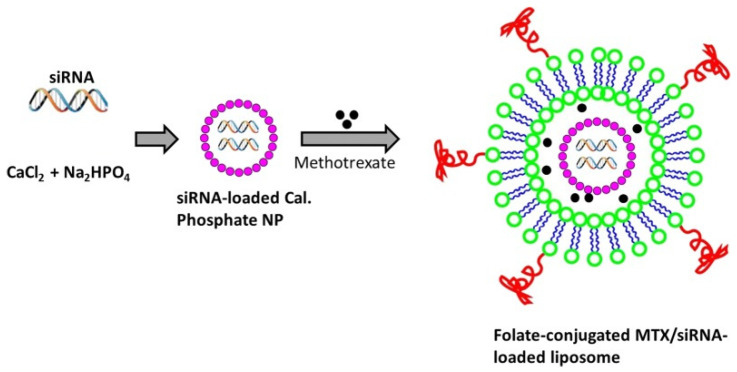
Schematic presentation of preparation of CaP/siRNA NP entrapped methotrexate loaded targeted liposome. Reprinted from ref. [81].

Methotrexate has been used in various polymeric structures with multiple formations for RA, although in some cases with low activity in the in vitro results, it shows perhaps its relatively unclear mechanism of action in RA treatment [77]. These results were observed in polysialic acid-trimethyl chitosan NPs loaded with MTX [78]. The NPs had good (10%) loading capacity, but with no significant change in the levels of IL-6 and IL-8 in the in vitro model of RA. However, in most cases, the in vitro results are positive and correlate with the in vivo. In one of them [79], NF-κB decoy oligonucleotide (ODN) was electrostatically adhered to the surface of polysialic acid-trimethyl chitosan, loaded with MTX. NF-κB ODNs have the ability to reduce inflammatory markers expression, in RA, by preventing nuclear translocation and expression of relative genes [80]. The ODN-coated NPs with MTX had satisfactory cellular uptake, as was observed by fluorescence microscopy, and were tested in vitro on two cell lines (SW982 cells and primary RASF cells) resulting in significant decrease in IL-6 and IL-8 in both models. The potential of NF-κΒ targeting was also shown by Duan and Li [81] who prepared folate conjugated liposomes loaded with MTX and NF-κΒ siRNA, for RA targeting. For their synthesis, calcium phosphate (CaP)/siRNA NPs were prepared, using pluronic F68 (Figure 3), free 1,2-Distearoyl-sn-glycero-3-phosphatidylcholine (DSPC), PEG bound DSPC and PEG-folate bound DSPC, were mixed with MTX in chloroform, and after the evaporation of the solvent, in the resulted folate conjugated liposome, the siRNA CaP complex was added filling the inner core of the liposomes (Figure 5).

Due to the folate receptor targeting, significant accumulation in activated macrophages was observed, blocking effectively the inflammatory signaling and with parallel sustained release even after 75 h. These effects led to very statistically significant (*p* < 0.001) paw thickness decrease, zero fluctuation of the arthritic and joint score, and substantial decrease in the inflammatory serum markers (Il-1β (interleukin-1β) and TNF-α). These results were also accompanied by low toxicity as the lymphocytes count dictates, further indicating the targeted delivery of the NPs.

In the area of oligonucleotides with MTX co-treatment, another effort [82] with si-RNA against Notch-1 mRNA, has been applied, using two diblock polymers [polycaprolactone-(PCL-PEG) and polyethylenimine-polyethylene glycol (PEI-PEG)]. PCL formed the inner hydrophobic core, and PEI and PEG, the outer, with PEI cationic surface influencing the binding of the anionic siRNAs. The two polymers were dissolved in tetrahydrofuran, where MTX was added and the final sonicated mixture, after the solvent evaporation, was mixed with the Notch-1 siRNA and incubated giving the final siRNA-MTX NPs. The NPs led to sustained release of MTX for more than 60 h and prolonged circulation, with significantly higher blood MTX concentration, compared to free-MTX, and substantial uptake by LPS activated cells, in vitro, with good cell viability even in high concentrations (100 μg/mL). The improved in vitro activity was shown and in vivo with the NPs to be able to decrease the paw thickness and the arthritic score remarkably, compared to the less effective MTX.

**Figure 6:**
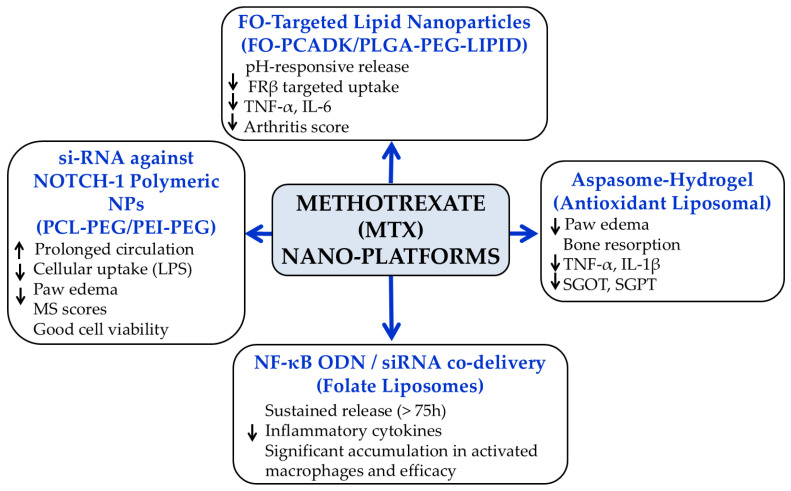
Methotrexate nano-formulations and their potential.

In another study [83], hydrogel MTX loaded, antioxidant liposomal aspasomes (AS) were prepared, comprised from carboprol, as gelling agent, for improved sustainability and effectiveness, for counteracting the low carrier efficiency of aspasomes, which are comprised of ascorbyl 6-palmitate, 1,2-dimyristoyl-sn-glycero-3-phosphocholine (DMPC) and cholesterol, in various ratios. The chosen formulation had smooth surface, but with increased (386.5 nm) particle size, drug entrapment of almost 20%, and steady 24 h permeation time. Transdermal application of the aspasome-hydrogel, in AIA model, in wistar rats, decreased the paw diameter, the bone resorption and the cartilage damage, significantly, with substantial decrease in the serum inflammatory and cachexia parameters [TNF-α, IL-β (interleukin-β), SGOT (serum glutamic-oxaloacetic transaminase), SGPT (serum glutamate pyruvate transaminase)], compared to the free-MTX group (Figure 6).

MTX, apart from FO, has also been used in co-treatment with other agents. In one of them Lutetium-177 was applied as a β-emitting radionuclide (for radiosynovectomy in RA), complexed with 1,4,7,10-Tetraazacyclododecane-1,4,7,10-tetra-acetic acid (DOTA) and hyaluronic acid (HA), used for targeting of CD44 and hyaluronan receptors, and bound on poly lactic-co-glycolic acid (PLGA) polymer [84]. The synthesis is rather interesting, starting with the mixing of PLGA with MTX in PVA solution, and after sonification and evaporation, the MTX-PLGA loaded NPs were activated in the presence of N-(3-dimethylaminopropyl)-N′-ethylcarbodiimide hydrochloride (EDC) and N-hydroxysulfosuccinimide sodium (NHS), for the incorporation of HA, using ethylene diamine as an intermediate linker. The final binding of DOTA-GGC (1,4,7,10-tetraazacyclododecane-N′,N″,N′′′-tetraacetic-Gly-Gly-Cys) was succeeded via the carboxylic activator HATU [O-(7-azabenzotriazol-1-yl)-1,1,3,3-tetramethyl uroniumhexafluoro phosphate]. The DOTA-HA-PLGA MTX loaded NPs were radiolabelled using ^177^LuCl_3_. The NPs had narrow distributed diameter of 167.6 nm with 95.2% encapsulation efficiency and 6% loading efficiency. The in vitro uptake by macrophages was eight-fold increased for the HA NPs than the non-HA NPs, that had a passive diffusion, whilst HA pre-incubation of the cells decreased significantly the penetration. The NPs had around 25% cell viability, perhaps related also to the proliferative activity of HA [85].

## 4. Inorganic Compounds and Enzymes

Selenium (Se) is an inorganic element with antioxidant properties and co-factor of various enzymes, like glutathione peroxidase, implicating in the function of the organism, as is the thyroid gland and the production of glutathione, and is a potential supplement for RA progression, whilst its low status seems to be a risk factor for the disease progression [86]. However, it can cause adverse effects depending on the administered form and dose. Dextrin stabilized Se NPs have been tested for their interaction with DNA and proteins, and their oral supplement was tested in chronic inflammatory arthritis, experimentally induced in wistar rats [87]. Wet chemical approach with the assistance of ascorbic acid was applied for the preparation of Se-NPs stabilized with 10% dextrin. The MTT cytotoxicity assay showed that dextrin coated Se-NPs (DSENPs) gave 75% cell viability compared to sodium selenite (5%) at 100 μg/mL, and at the same concentration or at 1000 μg/mL no interaction with the DNA or oxidative modifications were observed (perhaps due to sustained release of the Se content), whilst the interaction with the bovine serum albumin was time and concentration dependent. In vivo toxicity testing showed no abnormality concerning the clinical and histopathological characteristics of liver, spleen, and kidney of the animals. As for the arthritic study, rats treated with various doses ranging from 100–750 μg/kg of body weight, daily and orally, for 21 days, had significant difference than the arthritic group and in a similar manner with oral prednisolone at 10 mg/kg b.w. (body weight) at the 21st day. Furthermore, antioxidant enzymes levels tested in liver, kidney, and spleen were significantly improved in the DSENPs treated group whilst SOD and GPx levels were fully restored to normal levels. Similarly, C-reactive protein and prostaglandin E2 (PGE2) were decreased; however, in case of PGE2 levels, the decrease was not dose dependent, with the significant decrease observed at 100 mg/kg b.w. These results indicated the safe toxicological profile and the effective targeted antioxidant and anti-inflammatory potential of this selenium delivery method. Interestingly, the anti-RA effect was blocked when AMPK (AMP-activated protein kinase) inhibitor or palmitic acid where used, indicating that the activity is, at least partly, derived by targeting cell proliferation via mitochondria and lipogenesis.

Selenium has also been used, together with vitamin E succinate (VES), as ROS responsive polymeric structures for the release of berberine (an anti-RA potential agent targeting mitochondrial oxidative phosphorylation and lipogenesis [88] in RA therapy [89]). Vitamin E succinate–poly (lactic-co-glycolic acid)–selenocystamine dihydrochloride–methoxy poly(ethylene glycol) co-polymers (VES-PLGA-Se-Se-mPEG, VPseP) were prepared by VES activation with DCC and DMAP and the reaction of the final anhydrite with the hydroxyl group of OH-PLGA-COOH. In the second stage the carboxylic group of the ester was amidated with 2,2′-diselanediyldiethanamine and the product was also amidated with methoxyPEG-COOH in the presence of EDC and NHS as mediators. The final product was received and purified with co-precipitation and lyophilization. Berberine micelles were prepared by dialysis method, with ultrapure water, in sealed dialysis bag. The NPs had good average size (153 nm) with uniform spherical morphology, −5.12 mV zeta potential, more than 86% encapsulation efficiency and increased stability (more than two weeks). The polymeric micelles could passively diffuse to the tissues, but in inflamed areas the higher ROS production facilitated the collapse of micelles more than 80% faster than in the normal cells, and the cellular and mitochondrial uptake of nanostructured berberine was far more increased than berberine alone, and this effect explains the low oxygen consumption of the cells after the localization of berberine at mitochondria. These results led to the substantial attenuation of paw oedema, with similar effect to that of the methotrexate positive control group, accompanied by low joint bone destruction and cytokines generation, whilst berberine treatment was able to suppress only the inflammatory cytokines and not the clinical characteristics of the adjuvant induced arthritis. Selenium has been also used as a cleavable unit for stimuli responsive polymers. By using a diselenide group (easily cleaved to selenic acid at the oxidative conditions of RA), Wang et al. linked the hydrophobic cholesterol with the hydrophilic chondroitin sulfate that was assembled into NPs in an aqueous media [90]. For the synthesis, selenium was reduced to Na_2_Se_2_ in the presence of NaBH_4_ and then reacted with 2-chloroethylamine to give 2,2′-diselanediylbis(ethan-1-amine) that was amidated with chondroitin sulfate (CS) with the application of EDC/NHS. The resulted CS-SeSe was reacted with cholesteryl chloroformate in the presence of EDC/NHS giving the CS–SeSe–Chol after 24 h. This polymer encapsulated, by dialysis, the hydrophobic tofacinitib (Janus kinases inhibitor) and SP600125 (anthra [1,9-cd]pyrazol-6(2H)-one, c-Jun N-terminal kinases (JNKs) inhibitor) and lyophilized. The diameter of the NPs was 102.2 nm with drug loading and encapsulation efficiency of 12.6, 15.7, and 87.8, and 73.0%, respectively, for the two inhibitors, with 72 h stability with 7.4 pH and 10% fetal bovine serum solution, whilst in the presence of hydrogen peroxide, the release of both compounds is accelerating reaching up to 80%. This effect was observed also at the cellular uptake and drug release, in LPS stimulation, with increased internalization in case of inflammatory and oxidative conditions and offering decreased release in the non-inflammatory conditions. The application of the NPs on the LPS induced RAW264.7 cells showed a decrease in TNF-α, CD68, p-STAT1, and p-STAT3 expression. In vivo, in collagen induced arthritic mice, the NPs showed accumulation in the RA lesions and liver, with full elimination after 24 h and Cmax of 2 h. The treated group had very statistically improved arthritic score, paw thickness, and weight, with a notable decrease in pro-inflammatory markers expression and deterioration in the cartilage and joints, accentuating the multi-targeting effect of the combined active substances and the targeted delivery in the inflamed area by the resulted NPs.

An element with the ability to interconvert between the oxidized (^+4^) and the reduced (^+3^) form is cerium, and its oxides may be applicable as radical scavenging agents [91]. Cerium oxide nanoparticles due to increased surface can quench a wide number of radicals as in the oxidized form appears to exert catalase-like and SOD-like activity in their reduced form [92,93]. Thus, Weaver et al. [94] tried to incorporate cerium oxide nanoparticles (CONP) in alginate hydrogel for minimizing the phagocytosis and the cytotoxicity of the CONP. CONPs were prepared by dextran coating, in the presence of ammonium hydroxide, and further sonication and fabrication with alginate, by mixing with phosphate-buffered saline (PBS) solution in a capsule generator, and cross-linking with barium chloride. CONPs were able to preserve the metabolic integrity of murine beta cells, incubated with superoxide, significantly, in a dose dependent manner that leads to the metabolic activity and survival rate of the control group at 1.0 mM concentration of CONPs. However, at this concentration, the toxicity of the nanoparticles is evident due to internalization in the lysosomes. The dextran coating showed high retaining for over a year, scavenged hydrogen peroxide in a concentration dependent manner and did not cause cytotoxicity, since no cellular internalization was observed (even at 10 mM). However, increased cytoprotection was succeeded in superoxide incubated beta cells, an effect that endures for a long-term post-encapsulation, offering antioxidant protection against graft loss or auto-immune processes.

In the direction of SOD/Catalase mimetic procedure, hydrogen peroxide scavenging β-cyclodextrin (β-CD) material and SOD mimetic radical scavenger, tempol, were used for targeted therapy of inflammatory bowel disease [95] (2,2,6,6-Tetramethylpiperidin-1-yl)oxyl (TEMPO) and the 4-hydroxy derivative (TEMPOL), being aminoxyl radicals (nitrones), can efficiently trap ROS, generating stable nitroxyl radicals offering antioxidant potency, and anti-inflammatory, since minoxidil (MNX), a structurally similar molecule to nitrones have shown to stimulate the constitutive type of cyclooxygenase (COX-1) and inhibit the inducible COX-2, and the expression of TNF-a and NF-κB [96,97]. The ROS responsive material was prepared by 4-(hydroxymethyl) phenylboronic acid pinacol ester (PBAP) activation with CDI (N,N’-carbonyldiimidazole) and reacted with β-CD to give the NP material, that subsequently was loaded with tempol, with a nanoprecipitation-nano-assembly method [95]. The resulted NPs had narrow sized spherical shape, with hydrolysis potential in the presence of ROS, but no hydrolysis in the PBS incubation mixture, accentuating that the hydrolysis process takes place during oxidative conditions, eliminating the hydrogen peroxide and liberating the tempol molecules by the nanomatrix. The stability of NPs was shown in all the expected pH values of the GI system, and the accumulation at the colonic tissue of dextran sulfate induced acute colitis mice (after oral administration), in comparison to the normal mice, accentuates the targeted delivery of the NPs and their specific distribution as well, with notable mitigation of the relevant to colitis symptoms (as the disease activity index, the colon length preservation and the stained histological sections dictate) and reduced expression of pro-inflammatory (TNF-a, IFN-γ and IL-1β) and oxidative (MPO, MDA, superoxide production) mediators, with no safety concerns, such as death, toxic symptoms, or abnormal changes to the organs being aroused. Tempol has been also used, as a guest, in a host–guest interaction-based strategy, for the formation of affinity-controlled nanoparticles [98]. In this occasion, hydrophilic copolymers with polyethylene glycol and α-cyclodextrin or β-cyclodextrin were used. The first part consists of polyethylene glycol-b-poly(L-lysine) (PEG-PLL), which resulted by the reaction of PEG-amine with N-carboxyanhydride (NCA) of ɛ-(benzyloxycarbonyl)-L-lysine, and the final conjugates are derived by the reaction of 6-monotosyl α-cyclodextrin or β-cyclodextrin with the PLL block. The final host co-polymer could assemble tempol in spherical, inclusion complexed, particles with good loading ratio (0.25:1) and gradual release. The nanoparticles exhibited slight cellular toxicity, substantially reversed the phorbol 12-myristate 13-acetate oxidative stimulation in macrophages and the oxidative-induced cell-apoptosis, being more efficacious than the free tempol at the same dose. Furthermore, same as with the previous study [95], Tpl/PEG-P(b-CD) NPs dramatically decreased the levels of the inflammatory TNF-α, IL-1β, and IL-6, inhibiting the macrophage migration induced by OS or by the inflammatory monocyte chemoattractant protein-1 (MCP-1). In the same study, the effect of the NPs was also tested on dextran sulfate sodium (DSS) provoked ulcerative colitis in mice, with the NPs showing strong assembly at the site of the inflamed colonic tissues as the usage of a lipophilic fluorescent probe showed. Tempol NPs could significantly reverse the weight loss and the disease activity index (DAI), at dose 5 mg/kg, whilst tempol alone could offer comparable effect at dose 15 mg/kg. Additionally, haematological biomarkers levels were significantly improved and apart from the inflammatory markers levels, the oxidative stress parameters, like superoxide anion and malondialdehyde showed significant decrease, with histopathological improvement (improved abnormal variations of diseased colonic tissues, with microstructure similar to that of the normal group) as well. This may partly explain the anti-inflammatory and macrophages anti-migratory effects of tempol NPs, due to the direct interrelation between oxidant and inflammatory status.

Iron oxide nanoparticles seem to have potential applications for drug delivery, affecting catalysis reactions, treatment and imaging techniques [99,100,101]. Magnetic nanoparticles of dihydroascorbic acid (DHAA) with Fe_3_O_4_, covalently conjugated with hyaluronic acid (HA, hydrophilic polymer) and sulfasalazine [SSZ, disease-modifying anti-rheumatic drug (DMARD)] with anti-inflammatory and immunomodulatory potency] were prepared for the treatment of RA [102]. In this research, the core of DHAA-Fe_3_O_4_ was covered with HA in order to suppress its aggregation and it was anticipated that due to the increased ROS production and the high concentration of the hyaluronidase at the site of the inflammation, oxidative and HA breaking of the polymer conformation will occur. In view of these, DHAA and FeCl_3_ were synthesized via hydrothermal process and purified with external magnet application. At the second and third step, HA and SSZ were linked at DHAA-Fe_3_O_4_ with EDC and DMAP using water and DMSO, respectively, as solvents. The transformation of AA into DHAA, the capping effect of the later on the surface of iron oxide nanoparticles and the formation of DHAA–Fe_3_O_4_-HA-SSZ NPs were verified via Fourier Transform Infrared spectroscopy, and its magnetic properties were found to have saturation magnetization value of NPs 37 emu/g. The loading capacity of SSZ was 13.28% with dual curve release one at 130 h and one at 216 h, reaching up to 56%, with stable particle size as DLS and zeta potential analyses showed. The SSZ loaded NPs were tested in Freund’s Complete Adjuvant induced arthritis compared to SSZ, administered 14 days after RA induction and for 10 consecutive days. NPs showed approximately up to 75% decrease in Arthritis index and paw diameter, with very statistically significant decrease in TNF-α levels in the paw tissue, compared to the SSZ treated and the non-treated animals, with improved histological signs of tissue repair including the joints, the synovial folds and the articular surface of the respective bones and subjacent skin layers. Furthermore, normal thickness of the cartilage surface was recorded at the SSZ-NPs treated mice whilst there was distinct decrease in cartilage surface irregularity at the SSZ treated mice. These effects were accompanied by the very good toxicological profile of the loaded NPs, since the cardiac, liver, and kidney function (tested parameters blood urea nitrogen, urea, creatinine, creatinine kinase, and lactate dehydrogenase) remained unchanged with parallel significant decrease (*p* ≤ 0.001) in the levels of systemic inflammation, as the reduction in the C-reactive protein levels dictates, rendering stimuli-responsive delivery systems of SSZ loaded HA-coated magnetic nanocarriers as a promising tool for RA treatment.

Gold nanoparticles (AuNPs) have been introduced in the polymers field due to their facile synthesis and modification, with good compatibility and physical properties [103]. Gold-polydopamine (Au-PDA-NPs) (polydopamine-PDA, with excellent free radical scavenging properties [104]) nanoparticles, carrying tocilizumab (TCZ, a monoclonal antibody of Il-6), have been prepared using a direct reduction process of Au and ultrasonification for the preparation of the resulted NPs and mixing with TCZ solution [105]. The composite had a spherical size with the AuNPs binding on the PDANPs with good biocompatibility and promotion of the proliferation of chondrocytes and fibroblast with regenerating ability in case of Rheumatoid arthritis (RA), with further studies needed.

## 5. Clinical Translation, Regulatory Challenges and Safety Considerations

Despite the significant progress achieved in the development of antioxidant nanoformulations for autoimmune diseases, their translation from preclinical research to clinical application remains limited. Most currently available studies are based on in vitro, in cellulo, and animal models, which, although valuable for mechanistic understanding and proof-of-concept evaluation, often lack reproducing the complexity and heterogeneity of human autoimmune pathophysiology. Differences in immune responses, metabolism, disease progression, and nanoparticle biodistribution between animal models and humans may substantially affect therapeutic outcomes and safety profiles [106,107]. Consequently, only a limited number of nano-based formulations have progressed to clinical evaluation in autoimmune and inflammatory disorders.

One of the major challenges for clinical translation concerns the long-term safety and immunogenicity of nanoparticles. Although many polymeric systems, such as PLGA, PEG, chitosan, and hyaluronic acid derivatives, are generally considered biocompatible and biodegradable, their repeated administration may lead to unexpected immune activation, complement activation-related pseudoallergy (CARPA), accelerated blood clearance phenomena, or chronic tissue accumulation [108,109]. Particular concerns also arise regarding the interaction of nanoparticles with the mononuclear phagocyte system, liver and spleen accumulation, and the possibility of oxidative or inflammatory alterations induced by degradation products or inorganic nanoparticle components [110]. Furthermore, the physicochemical properties of the nanoparticles, including particle size, surface charge, hydrophobicity, and coating materials, may significantly influence cellular uptake, circulation time, immunogenicity, and toxicity [111].

Another important limitation involves manufacturing reproducibility and large-scale production. Many nanoformulations are synthesized under highly controlled laboratory conditions using multistep preparation methods that are difficult to be standardized and reproduced at industrial scale level, with variability may affecting nanoparticle size distribution, drug loading efficiency, stability, release kinetics, and biological activity, thereby complicating quality control and regulatory approval procedures [112]. In addition, the sterilization, storage stability, and scalability of complex multifunctional nanoparticles remain unresolved issues for many experimental systems [113,114]. Regulatory approval also represents a substantial barrier to the clinical implementation of nanomedicines. Regulatory agencies still face challenges in establishing harmonized guidelines specifically tailored to nanotherapeutics and nanoformulations. The complexity of nanomaterials often makes conventional pharmacokinetic and toxicological evaluation insufficient, requiring additional characterization regarding biodistribution, degradation, immunotoxicity, and long-term environmental and biological impact [115]. Moreover, the absence of universally accepted standards for nanoparticle characterization and biological evaluation contributes to delays in regulatory assessment and approval processes [116].

Clinical efficacy may also be influenced by the highly heterogeneous nature of autoimmune diseases. Variability among patients regarding genetic background, inflammatory status, disease stage, microbiome composition, and oxidative stress burden may alter therapeutic responsiveness to nano-based systems [117]. Therefore, future therapeutic approaches may require personalized or precision nanomedicine strategies, combining biomarker-guided patient stratification with adaptable nanoparticle platforms capable of individualized targeting and drug release profiles. Importantly, although several nanomedicines have already received approval for oncological and infectious diseases, relatively few have entered clinical trials for autoimmune disorders [118]. Thus, although various approaches, as those described in this review, have shown encouraging preliminary results, particularly regarding reduced systemic toxicity and improved tissue targeting; however, large randomized clinical studies remain scarce, and additional long-term clinical investigations are, therefore, necessary to validate efficacy, pharmacokinetics, safety, immunogenicity, and patient tolerability in chronic autoimmune conditions.

## 6. Future Perspectives and Conclusions

**Figure 7:**
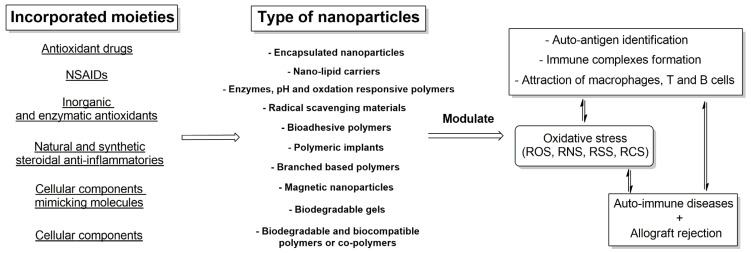
Nanoparticles incorporating antioxidant, anti-inflammatory and cellular components for the modulation of autoimmune diseases and allograft rejection.

Autoimmune diseases (AIDs) still pose a significant challenge due to their complex etiology, chronic progression, and the intriguing balance between immune suppression and preservation of essential immune functions. The interrelation between oxidative stress, inflammation, and immune dysregulation constitutes a vicious cycle that not only accentuates tissue damage but also complicates therapeutic interventions. The investigation for nanoparticles, polymeric (PNs) and non-polymeric, as drug delivery systems offers a promising approach to enhance the efficacy and safety of antioxidant and anti-inflammatory therapies for AIDs. The evidence in the reviewed literature suggests that nanoparticles can improve pharmacokinetics, enhance bioavailability, and allow the precise targeting of inflammatory and immune pathways, while simultaneously reducing systemic toxicity and adverse effects commonly related to conventional immunosuppressants (Table 1). Moreover, their ability to encapsulate both organic and inorganic antioxidant compounds, enzymes, or synergistic drug combinations position them as multi-purpose platforms in the evolving landscape of nanomedicine (Figure 7).

Looking to future research, it should focus on several critical areas, such as the deeper understanding of the pathophysiological mechanisms specific to each autoimmune condition, which is essential for the rational design of disease-specific nanocarriers. The development of smart, stimuli-responsive nanoparticles that can release therapeutic agents in response to specific intracellular triggers (e.g., ROS levels, pH changes) may further improve targeted delivery strategies. These options together with the integration of artificial intelligence (AI) and machine learning could enable predictive modeling for nanoparticle design and individualized therapy planning.

In conclusion, while the field of antioxidant nanoformulations for autoimmune diseases seems very promising and fruitful, sustained interdisciplinary efforts will be necessary to bridge the gap from bench to bedside. Addressing current limitations through technological innovations, rigorous safety evaluations, and patient-centered research will ultimately determine the success and real-world impact of these novel therapeutic platforms, with substantial scientific, manufacturing, regulatory, and clinical challenges being important to be addressed before their widespread clinical adoption becomes feasible.

## Figures and Tables

**Table 1:** Antioxidant Polymeric and Non-Polymeric Nanoformulations for Autoimmune Diseases.

Type of Polymer Carrier	Drug	Pharmacokinetic Advantages	Efficacy Findings
Aminomethacrylate/succinylated β-cyclodextrin nanogels with PLA-PEG copolymer and liposomes	Mycophenolic acid (MPA)	Improved solubilization, APC internalization [22], targeted CD4^+^ T-cell delivery [23], enhanced loading and stability [23].	Increased survival for systemic lupus erythematosus treatment in mice [22].
PEG-PLGA nanoparticles	Mycophenolate mofetil (MMF)	Controlled delivery and improved endothelial uptake [24].	Reduced transplant vasculopathy and preserved heart histology post-transplantation [24].
Eudragit S100	Metronidazole (MTZ)	Colon-targeted release, improved encapsulation efficiency and stability [26].	Suitable sustained release for IBD treatment [26].
p(DapMA) homopolymer and HEMA-DapMA copolymer	Dapsone (DAP)	High stability with low hydrolysis rate of DAP; improved solubility, sustained release, improved distribution [27].	Dose-dependent NO inhibition and anti-inflammatory activity [27].
Nanolipid carriers (NLCs)	Teriflunomide (Ter)	Enhanced intranasal permeability, mucoadhesion, sustained release [32].	Improved demyelination and behavioral outcomes in MS model [32].
Hydroxyapatite (HAP) nanoparticles	Ter + Methotrexate	Sustained release and enhanced drug loading [33].	Improved rheumatoid arthritis joint architecture and arthritis scores [33].
Poly(DL-lactide) (PDL 02) with sesame oil (SO) system	Tenoxicam	Reduced burst release and prolonged release [37].	Reduced paw edema and improved oxidative biomarkers (SOD, MDH, MPO) in arthritis [37].
Poly(lactic acid) nanoparticles	Betamethasone	Retinal/choroidal retention and sustained delivery [39].	Reduced uveitis clinical and histological scores [39].
Carboxyl-terminated PLGA nanoparticles	Dexamethasone	Sustained release for >3 weeks after subconjuctival administration [40].	Reduced retinal microglial activation and experimental autoimmune uveoretinitis severity [40].
PEG-b-PCL copolymer micelles	Celastrol	Improved aqueous solubility and local ocular delivery [41].	Reduced cytokines, TNF-α, and VEGF expression by the macrophages with reduced corneal neovascularization [41].
Eudragit RS100	Dexamethasone	Enhanced epidermal targeting and high encapsulation efficiency [43].	Improved skin delivery for dermatologic autoimmune diseases [43].
HPMA copolymer	Dexamethasone	pH-sensitive release at inflamed RA synovium [44].	Prolonged anti-inflammatory activity and cartilage preservation [44].
Tannic acid/Polyethylene glycol (PEG) or Pluronic (F68/F127) copolymers	Dexamethasone	Esterase-triggered release, ROS scavenging, GI stability [47].	Improved colitis symptoms, reduced myeloperoxidase and TNF-α [47].
Dendrimeric polymer	PAMAM-G3	Scavenging extracellular nucleic acids and TLR inhibition [56]; improved arthritis tissue targeting and DNA binding [56].	Reduced lupus severity, kidney injury, and autoantibody levels [56]; reduced TNF-α and IFN-α improved joint pathology in RA [56].
Polyplex-like nucleic acid-peptide assemblies	GpG oligonucleotide	Protection from enzymatic degradation and tunable properties [66].	Reduced TLR signaling and myelin-driven inflammation in MS [66].
Poly(β-amino ester) (Poly1)	GpG oligonucleotide	Sustained nucleic acid release over several days [68].	Reduced IL-6 and promoted tolerogenic T-cell phenotype [68].
Lipid-polymer	MTX	pH-responsive release and FRβ macrophage targeting [75].	Reduced RA clinical scores and inflammatory cytokines [75].
PAMAM dendrimers	MTX	FRβ-mediated targeting and multivalent loading [76].	Improved RA histological and clinical outcomes [76].
Polysialic acid-trimethyl chitosan	NF-κB decoy oligonucleotide (ODN) + MTX	Improved MTX loading and enhanced cellular uptake and dual targeting [80].	Reduced IL-6 and IL-8 expression in RA cells [80].
Folate-conjugated PEGylated liposomes	MTX	Sustained release (>75 h) and activated macrophage targeting [81].	Strong reduction in arthritis severity and inflammatory cytokines such as Il-1β and TNF-α [81].
PCL-PEG and PEI-PEG	MTX	Sustained MTX release and prolonged circulation [82].	Reduced paw thickness and arthritis score [82].
Carbopol hydrogen with liposomal aspasomes	MTX	Improved transdermal permeation and sustainability [83].	Reduced paw edema, bone resorption, and cytokines such as TNF-α and IL-β [83].
Hyaluronic acid (HA)-coated PLGA nanoparticles	MTX	Enhanced macrophage uptake and radiolabel targeting [85].	Improved RA pathology and inflammation [84].
Dextrin nanoparticles (NPs)	Selenium	Sustained selenium release and reduced oxidative interaction [87].	Restored antioxidant enzymes and improved arthritis markers [87].
VES-PLGA-Se-Se-mPEG copolymer	Selenium + vitamin E + berberine	ROS-triggered release and mitochondrial targeting [88].	Reduced paw edema and inflammatory cytokines [88].
Chondroitin sulfate-diselenide-cholesterol polymer	Selenium + Tofacinitib + SP600125	Oxidative-triggered release and RA lesion accumulation [90].	Improved arthritis score and reduced cytokine expression [90].
Alginate hydrogel	Cerium oxide	Long-term retention and reduced phagocytosis [94].	Protected beta cells from oxidative injury [94].
β-cyclodextrin ROS-responsive polymer	Tempol	ROS-triggered hydrolysis and color targeting [98].	Reduced colitis severity and oxidative markers such as TNF-a, IFN-γ and IL-1β [98].
Fe_3_O_4_/Humic Acid (HA)	Sulfasalazine	ROS/hyaluronidase-responsive release and magnetic properties [102].	Strong reduction arthritis index and TNF-α [102].
Gold-polydopamine nanoparticles (AuNPs)	Tocilizumab	Improved nanoparticles stability and regenerative support [105].	Promoted chondrocyte and fibroblast proliferation in RA [105].

## Data Availability

No new data were created or analyzed in this study. Data sharing is not applicable to this article.
